# Urgent Abdominal Re-Explorations

**DOI:** 10.1186/1749-7922-1-10

**Published:** 2006-04-04

**Authors:** Haluk Recai Unalp, Erdinc Kamer, Haldun Kar, Ahmet Bal, Mustafa Peskersoy, Mehmet Ali Onal

**Affiliations:** 1 General Surgeon. Department of General Surgery, 4^th ^General Surgery Clinic. Izmir Ataturk Training and Research Hospital, Izmir, Turkey; 2 General Surgeon, Associated Chief Department of General Surgery, 4^th ^General Surgery Clinic. Izmir Ataturk Training and Research Hospital, Izmir, Turkey; 3 General Surgeon, Chief. Department of General Surgery, 4^th ^General Surgery Clinic. Izmir Ataturk Training and Research Hospital, Turkey

## Abstract

**Background:**

Treatment of a number of complications that occur after abdominal surgeries may require that Urgent Abdominal Re-explorations (UARs), the life-saving and obligatory operations, are performed. The objectives of this study were to evaluate the reasons for performing UARs, outcomes of relaparotomies (RLs) and factors that affect mortality.

**Methods:**

Demographic characteristics; initial diagnoses; information from and complications of the first surgery received; durations and outcomes of UAR(s) performed in patients who received early RLs because of complicated abdominal surgeries in our clinic between 01.01.2000 and 31.12.2004 were investigated retrospectively. Statistical analyses were done using the chi-square and Fisher exact tests.

**Results:**

Early UAR was performed in 81 out of 4410 cases (1.8%). Average patient age was 50.46 (13–81) years with a male-to-female ratio of 60/21. Fifty one (62.96%) patients had infection, 41 (50.61%) of them had an accompanying serious disease, 24 (29.62%) of them had various tumors and 57 (70.37%) patients were operated under emergency conditions during first operation. Causes of urgent abdominal re-explorations were as follows: leakage from intestinal repair site or from anostomosis (n:34; 41.97%); hemorrhage (n:15; 18.51%); intestinal perforation (n:8; 9.87%); intraabdominal infection or abscess (n:8; 9.87%); progressive intestinal necrosis (n:7; 8.64%); stomal complications (n:5; 6.17%); and postoperative ileus (n:4; 4.93%). Two or more UARs were performed in 18 (22.22%) cases, and overall mortality was 34.97% (n:30). Interval between the first laparotomy and UAR averaged as 6.95 (1–20) days, and average hospitalization period was 27.1 (3–78) days.

Mortality rate was found to be higher among the patients who received multiple UARs. The most common (55.5%) cause of mortality was sepsis/multiple organ failure (MOF). The rates for common mortality and sepsis/MOF-dependent mortality that occured following UAR were significantly higher in patients who received GIS surgery than in those who received other types of surgeries (p:0.000 and 0.010, respectively).

**Conclusion:**

UARs that are performed following complicated abdominal surgeries have high mortality rates. In particular, UARs have higher mortality rates following GIS surgeries or when infectious complications occur. The possibility of efficiently lowering these high rates depends on the success of the first operations that the patient had received.

## Introduction

The term "Relaparotomy" (RL) refers to operations performed within 60 days in association with the initial surgery. RL is categorized as early or late; radical or palliative; urgent or elective; and, planned or unplanned depending on the performed period, its purpose, urgency, and whether or not it is scheduled, respectively [1]. Urgent abdominal re-explorations (UARs) following complicated abdominal surgeries are generally known as "final-choice operations" with high mortality and morbidity rates [2].

This retrospective study aimed to determine the incidence, causes and outcomes of UARs, identify factors that affect mortality and describe outcomes of multiple RLs performed between 2000–2004 in Ataturk Training and Research Hospital, Izmir, Turkey.

## Methods

In this study, 81 UAR cases (out of 4410 patients who underwent abdominal surgeries between 01.01.2000 and 31.12.2004) were documented through scanning hospital archives for their age, sex and initial diagnoses; pre- and per-operative findings; surgical procedures applied during, and postoperative complications occured following the first operation; and for UAR intervals and outcomes. Mortality rates and reasons following UARs were also investigated.

Patients who received damage control surgeries, planned RLs, minimally invasive surgery as percutaneous drainage and those who received surgical interventions on the abdomen wall for the treatment of evisceration/eventration were excluded in order to homogenize the study group.

Complications were determined by performing hematological and radiological examinations upon observation of patient's altered general condition or detection of existence of blood or inflammatory material or intestinal content causing treatment-resistant peritonitis in the drain in postoperative period.

The following parameters were considered as urgent laparotomy decision criteria: i) existence of hemorrhage resistant to medical treatment, ii) existence of progressive peritonitis, iii) existence of abscess where percutaneous drainage is either impossible or ineffective, iv) continuous contamination of abdominal cavity with fecal content, v) existence of necrosis, vi) existence of ileus resistant to decompression or medical treatment, vii) worsening of patient's clinical condition despite medical treatment.

Initial operations were performed by assistant general surgeons instructed by specialized general surgeons while all UARs were performed by specialized general surgeons. It was determined that all patients received a 2nd generation cephalosporin prophylaxis prior to surgery and no additional antibiotics were administered unless a complication was observed in postoperative period. Suitable treatment regimens, durations of which averaged 12 (3–29) days, were used in accordance with culture/antibiogram results in patients who required additional antibiotics (n:61/81, 75.30%) following having received UARs. Standard colon emptying was performed in non-urgent cases.

Statistical analyses were done using *chi-square *and *Fisher exact tests *by an independent comparisons among groups by a statistician who was blinded to the study. A *p *value of < 0.05 was considered as significant.

## Results

UARs were performed for 121 times in 81 (1.8%) patients out of 4410 abdominal surgery cases. Number of male and female patients was 60 (74.07%) and 21 (25.93%), respectively. Their ages averaged 50.46 (13–81) years. Fifty seven (70.37%) patients were operated under emergency conditions, 51 (62.96%) had infection*s*, 24 (29.62%) had tumors and 41 (50.61%) patients had accompanying serious diseases at the time of the first surgery.

UARs were performed most commonly following lower gastrointestinal system surgeries (Table 1). The most common cause of UARs was leakage from intestinal repair site or anostomosis (n:34, 41.97%), and the most common mortality reason was sepsis and multiple organ failure (MOF) (Table 2). Mortality rate following UARs was found as 37.03% (n:30).

**Table 1:** First surgical interventions that require RL

**Reason for the first laparotomy**	**N (%)**
Lower GIS	28 (34.56)
-Colon and rectum	15 (18.51)
-Small intestine	10 (12.34)
-Appendix	3 (3.70)
Upper GIS*	23 (28.39)
-Stomach	16 (19.75)
-Duodenum	9 (11.11)
HPB** system	8 (9.87)
-Liver	3 (3.70)
-Gall Bladder	3 (3.70)
-Pancreas	2 (2.46)
Multi-organ	5 (6.17)
Vascular	4 (4.94)
Gyneco-pathological interventions	4 (4.94)
Strangulated umbilical/ventral hernia	3 (3.70)
Peritonitis	3 (3.70)
Spleen	3 (3.70)

* Gastrointestinal system ** Hepato-pancreatico-biliary

**Table 2:** RL reasons and mortality rates

RL* reason	**n (%)**	**Mortality n (%)**	**Cause of Mortality**	**n**
Intestinal content	42 (51.85)	19 (45.23)		
- Intestinal repair site or anastomosis failure	34 (41.97)	14 (41.17)	Sepsis and MOF	9
			Cardiac	2
			Respiratory	1
			Thromboemboli	2
- Intestinal perforation	8 (9.87)	5 (62.50)	Sepsis and MOF	4
			Respiratory	1
Hemorrhage	15 (18.51)	3 (20)	Coagulopathy	2
			Hypovolemic shock	1
Intraabdominal infection or abscess	8 (9.87)	1 (12.50)	Sepsis and MOF	1
Intestinal necrosis	7 (8.64)	6 (85.71)	Thromboemboli	4
			Sepsis and MOF	2
Stomal complications	5 (6.17)	-		
Ileus	4 (4.93)	1 (25)	Respiratory	1

* Relaparatomy

UAR was conducted once and twice or more in 63 (77.77%) and 18 (22.22%) patients, respectively, and average number of re-exploration was 1.49. Mortality rate was 28.57% (n:18) and 66.66% (n:12) in patients who received UAR once and for multiple times, respectively (p = 0.102). Table 3 describes demographics of patients as well as statistics regarding mortality rates.

**Table 3:** Patient demographics and features of surgical interventions

		n (%)	Mortality n (%)	Statistics
Sex	Male	60 (74.07)	23 (38.33)	p: 0.884, OR*:0.80
	Female	21 (25.92)	7 (33.33)	0.24<%95CL**>2.53
Surgery	Urgent	57 (70.37)	25 (43.85)	p:0.087, OR:0.34
	Elective	24 (29.62)	5 (20.83)	0.09<%95CL>1.12
Infection in first operation	Yes	51 (62.96)	19 (37.25)	p:0.852, OR:0.98
	No	30 (37.03)	11 (36.66)	0.34<%95CL>2.72
Tumor	Yes	24 (29.62)	6 (25)	p:0.228, OR:2.18
	No	57 (70.37)	24 (42.10)	0.69<%95CL>7.68
Accompanying disease	Yes	41 (50.61)	18 (43.90)	p:0.286, OR:0.55
	No	40 (49.38)	12 (30)	0.22<%95CL>1.50
Number of UARs***	One	63 (77.77)	18 (28.57)	p:0.007, OR:5
	Multiple	18 (22.22)	12 (66.66)	1.44<%95CL>18.50

* Odds Ratio ** Confidence limit *** Urgent abdominal re-explorations

Mortality rates following UARs in GIS surgeries (n:27/51, 52.94%) were significantly higher compared with those in other types of surgeries (n:3/30, 10%) (p:0.000, OR:0.10, 0.02<%95CL<0.39). Similarly, the rates for common mortality and sepsis/MOF-dependent mortality that occured following UAR were significantly higher in patients who received GIS surgery (n:15/51, 29.41%) than in those who received other types of surgeries (n:1/30, 3.33%) (p:0.010, OR:0.08, 0.00<%95CL<0.61). No statistically significant difference (p:0.855, OR:0.95, 0.27<%95CL<3.28) was found between mortality rates following lower GIS surgeries (n:15/28, 53.57%) and those following upper GIS surgeries (n:12/23, 52.17%). Similarly, no statistically significant difference (p:0.161, OR:0.33, 0.06<%95CL<1.40) was found between mortality rates in lower GIS surgeries (n:11/28, 39.28%) and those in upper GIS surgeries (n:4/23, 17.39%) following RLs due to sepsis/MOF (Table 4).

**Table 4:** Surgery and Mortality Reasons

	n (%)	**Mortality reason**				
		Sepsis/MOF*	Thromboemboli	Respiratory	Cardiac	Hemorrhage
Lower GIS**	28 (34.56)	11	1	2	1	-
Upper GIS	23 (28.39)	4	5	1	1	1
HPB***	8 (9.87)	-	-	-	-	1
Multiorgan	5 (6.17)	1	-	-	-	-
Vascular	4 (4.94)	-	-	-	-	1

***Multiorgan Failure * Gastrointestinal system ** Hepato-pancreatico-biliary

Average age of patients who died after early UAR was 52.16 (range 17–81), while that of surviving patients was 49.47 (range 13–81). Average interval between the first operation and UAR, and average hospitalization time was 6.95 (range 1–20) and 27.1 (range 3–78) days, respectively.

## Discussion

The incidence of urgent relaparotomy-requiring complications has been reported as 1–4.4% in patients who underwent abdominal cavity/organ-related surgeries [2-8]. Consistent with previous studies, we here report a UAR ratio of 1.80% in the same type of patients.

RL-requiring complications can be categorized into 5 groups: (i) hemorrhage into intestinal canal or abdominal cavity (ii) peritonitis that occurs in the absence or presence of a perforation (iii) mechanical or paralytical postoperative ileus (iv) eventration or evisceration (v) miscellaneous complications [2,9]. The incidence of UAR-requiring complications varies depending on disease characteristics of patients hospitalized, and types of surgeries they had received [3]. RLs were reported to be performed following diffuse or limited peritonitis; ileus; eventration; hemorrhage; and other causes in 32–51.31%; 25–62.79%; 7.23–22%; 3.3–19%; and in 2–3.28% of the patients, respectively [8,9]. In our study, UARs were most commonly (n:42/81, 51.85%,) performed for controlling release of intestinal content into abdominal cavity (leakage from intestinal repair site or anostomosis: n:34/81, 41.97%; and intestinal perforation: n:8/81, 9.87%). Other common causes were hemorrhage (18.51%); intestinal perforation (9.87%); and intraabdominal infection or abscess (9.87%). Regardless of their incidence, these complications had a life-threatening and UAR-requiring nature in common. Hence, immediate diagnoses of complications and urgent intervention by performing RLs could save many lives [2]. In spite of early diagnosis possibilities and therapeutical progress, mortality rates following UARs are still high ranging from 15.5% to 61.5% depending on the severity of complications [3,5,8,10,11]. We here report a mortality rate of 37.03% following UARs.

One of the most important factors affecting mortality rates in UAR is the cause of RL. Wound separation and obstruction, hemorrhage and infection, and anostomosis failure have low, mild, and high mortality risks, respectively [2]. In our study, we have determined that mesentery artery embolus, intestinal perforation and anastomosis failure had high mortality rates, while mortality rates following obstruction and hemorrhage, and intraabdominal infection and abscess were mild and low, respectively. Another important factor affecting mortality is the organ/system that relaparotomy is performed on. Consistent with this view, as has been shown in our study, mortality rate is higher in GIS surgery particularly because of greater septic complication rates following UARs.

Although different surgery centers reported different values, there is a common consensus regarding the most common cause of urgent RLs, consistent with our study, as inflammatory complications [4,12-14]. Immediate diagnosis (ideally within the first 36 hours of operation) and early surgical intervention accompanied by conventional treatments have been shown to reduce mortality by removing the infectious focal from the body and ameliorating metabolic problems [2,7,10,13]. Determining the focus of sepsis, however, may not be possible in all cases. The ratio of septic focal determination was reported as 17% by Hutchins et al [7]. On the other hand, removal of all of the determined septic foci by surgical intervention may not always be possible. Mulier et al, showed the existence of residual peritonitis in 9% and 41% of purulant/biliary and feacal peritonitis cases who underwent urgent surgeries for controlling the source of infection, respectively [14]. These findings may partly explain the reason why sepsis persists following re-explorations conducted for treatment of infective complications. In our study, the most common cause of mortality has been found as sepsis and MOF secondary to sepsis (n:20, 55.55%). Other common causes are thromboemboli 16.66% (n:6); respiratory reasons 11.11% (n:4); cardiac reasons 8.33% (n:3); and hemorrhage 8.33% (n:3).

Mortality rate following a single RL and multiple RLs was 30.6% and 65.6%, respectively, confirming the positive correlation between the number of RLs and mortality rate [10]. Similarly, we found high mortality rates in patients who received multiple RLs. This increased rate of mortality following multiple RLs might be caused by residual infections, inadequately treated previous complications or novel complications following RLs and the reduction in patient's reserves. This hypothesis is further supported by the fact that mortality rates are higher in older patients who have multiple organ failures [2,10,15]. The reason for the higher mortality rates in patients who underwent multiple RLs depends on the etiology, but not the number of RLs. Delayed surgical intervention for treatment of an intraabdominal septic focus might cause sepsis and, hence, mutiorgan failure. Studies have shown that early diagnosis following the first abdominal surgery and management by early RL of intraperitoneal sepsis decrease multiorgan failure by 60% and, thereby, lowers mortality rates [7]. Mortality rate following re-exploration in cases in whom treatment-resistant sepsis was identified 37.5%, whereas this ratio was 67% in patients who did not receive re-exploration suggests that the surgeon should seriously consider performing a RL [16]. On the other hand, there's no consensus about when and how to intervene septic complications of abdominal surgeries. The common sense in the literature persuades the surgeon to perform UARs "with the right timing" [4]. Mortality rates were lowered from 46% to 20.5% and from 21.4% to 15.3% in immediately diagnosed cases who underwent UARs with the right timing in the studies of Desiaterik et al, and Zavernyi et al, respectively [11,17]. However, since the "right timing" varies from patient to patient, "the experience of the surgeon" might be one of the factors that reduce the mortality rate.

Another early RL-requiring reason following abdominal operations is postoperative hemorrhage. These hemorrhages can originate from the drain and the incision line, or can present as upper or lower GIS hemorrhages. However, a serious postoperative follow-up is required in order to determine the origin of intraperitoneal hemorrhages. Postoperative hemorrhage rate following abdominal surgical interventions is 0.1% [18]. This low rate is a result of adequate and appropriate preoperative preparation and early diagnoses of patients who are under hemorrhage risk. However, it has been shown that 22.22% of the RL-requiring hemorrhages were observed in patients who were operated under elective conditions and that 72.22% of the hemorrhages in these cases were caused by technical mistakes (such as inadequate hemostasis) in the first operation [18]. Postoperative minor hemorrhages could be followed-up conservatively. However, patients who require recurrent blood transfusions because of long-lasting hemorrhages may develop disseminated intravascular coagulopathy. Abundant hemorrhages in postoperative period may abolish patient's clinical condition; it may also be difficult to diagnose hemorrhages into GIS in the early period in spite of the existence of a drain [19]. Mortality rate in postoperative hemorrhages varies from 18.4% to 33.33% depending on delays in diagnosis [19,20]. Mortality rates are higher particularly after urgent laparotomies and in geriatric patients [15]. In our study, we found the RL-requiring hemorrhage rate in postoperative period as 0.34%, while the mortality rate in hemorrhage was found as 20%. Abundant hemorrhage following coagulopathy were found in two out of three patients who died, while the other patient died because of thromboembolic complications during RL that was performed for treating hemorrhage.

The most common cause of obstructions in postoperative period is adhesive lesions which is one of the common problems in general surgery [6,20]. The risk of adhesions exists throughout the lifetime following laparotomies. Rate of early ileus following abdominal operations is 0.86% [21]. However, difference approaches for the treatment of early ileus exist among surgical centers. Thus, UAR-requiring cases because of intestinal obstructions vary in a broad scale between 1/4 and 2/3 of all UARs [8,9]. Immediate diagnosis, intubation of GIS and efficient decompression are required for a better prognosis in patients who developed intestinal obstruction [6]. However, it is difficult to decide when to operate the patients whose clinical condition did not improve in time. Ellozy et al, suggested that an immediate operation should not be considered in these patients since 87% of postoperative small bowel obstructions can be reversed by nasogastric decompression [22]. In our study, 4 cases (4.93%) who received postoperative early ileus diagnosis and who did not respond positively to medical treatment received UAR on the 4th day on an average (3th, 4th, 4th and 5th days). Obstruction was brid in 3 (75%) out of these four patients who survived following RL. The fourth patient who received a RL on the 5th day required a broad resection because of intestinal torsion, and this patient died in postoperative period. Postoperative ileus might not have recovered and a large intestinal segment necrosed during surgical exploration might have observed as in the case with this patient, although the patient received identical decompression and medical treatment with what the other three patients have received. However, that one out of every three patients who did not respond positively to conservative surgery can die in postoperative period should be kept in mind [21,23]. On the other hand, surgery performed earlier than it is required may be risky for patients who are likely to respond positively to medical treatment.

That such pulmonary, renal or thromboembolic complications, or wound infections are observed more commonly in patients operated for neoplastic reasons than in those operated for non-neoplastic reasons is well documented. However, since only "urgent surgery-requiring" complications have been evaluated in our study, a statistically significant difference in terms of mortality between UARs in patients operated for malign reasons and those in patients operated for benign reasons have not been found. Similarly, gender, existence of infection during the first operation, performance of surgery under urgent conditions or accompanying diseases did not influence mortality following RL in our study.

In conclusion, we suggest that the most efficient way of reducing UAR and mortality rates is actually "avoiding the possible complications during the first surgery". On the other hand, the success of the surgeon would be proportionate to his correct responses to such questions as "to whom, when, under what conditions, why and how the surgery should be conducted" when UAR is required.
